# Cytotoxicity of Diimine Palladium (II) Complexes of Alkyldithiocarbamate Derivatives on Human Lung, Ovary and Liver Cells

**Published:** 2012

**Authors:** Narges Aryanpour, Hassan Mansouri-Torshizi, Maryam Nakhjavan, Farshad H. Shirazi

**Affiliations:** a *Department of Toxicology and Pharmacology, School of Pharmacy, Shahid Beheshti University of Medical Sciences, Tehran, Iran. *; b *Department of Chemistry, Faculty of Science, University of Sistan and Bluchestan, Zahedan, Iran. *; c *Pharmaceutical Sciences Research Center, Shahid Behesthi University of Medical Sciences, Tehran, Iran.*

**Keywords:** Palladium complex, Dithiocarbamates, Cytotoxicity, Liver cells

## Abstract

Three new Complexes of formula [pd(bpy)(R-NH-CSS)] Cl (where bpy is 2/2′- bipyridine, and R-NH-CSS is butylamine, hexylamine- and octyamine-dithiocabamate anion) have been synthesized by University of Sistan and Blachostan. These complexes have been characterized by spectroscopic methods such as ultraviolet-visible, infrared and ^1^H-NMR as well as conductivity measurements and chemical analysis. In these complexes, each of the dithiocarbamate ligands coordinates to Pd (II) center as bidentate with two sulfur atoms. We have found a 1:1 electrolyte in water conductivity test for the above mentioned compounds. To measure the biologic activity and potential anticancer efficacy of these compounds, they have been compared with cisplatin and its palladium analogue of [Pd (NH_3_)_2_ Cl_2_] on three different cell lines of human hepatocarcinoma HepG2, human ovarian carcinoma OV2008, and human lung adenocarcinoma A549. Clonogenic assay has shown LD_50_s in the range of 0.131±0.025 to 0.934 ± 0.194 for these compounds on above cell lines. In comparison, cisplatin has shown LD_50_s of 0.838 ± 0.074, 2.196 ± 0.220, and 2.799 ± 0.733 on OV2008, HepG2 and A549 cell lines, respectively. As a conclusion, above three new complexes have shown higher cytotoxicities compared to cisplatin on three different human cell lines. Based on biological tests, these compounds may potentially be considered as good anticancer candidates for further pharmacological studies.

## Introduction

Currently, cis-diamminedichloroplatinum (II) (cisplatin) is being used as an anticancer drug against several human cancers, such as: testicular cancer, Ovarian and bladder cancer, osteogenic Sarcoma, head and neck cancer, endometrial and cervical cancer and non- small cell lung cancer (1). Due to the frequent resistant to this drug during cancer chemotherapy (2), as well as dose limiting toxic side effects of platinum anticancer drugs, there have been many attempts to find complexes with greater potency and less toxicity than the existing clinical drugs. As a consequence of this, attention has naturally turned to the other platinum group metals, ruthenium, rhodium, palladium, osmium and iridium (3). Among them, preparation and study of cis-dichloropalladium (II) and other closely related complexes having chelating ligands such as *N-N*-diamines, *N,S*-amino–thioether, diaminoacids, dicarboxylic acids and dithiocarbamates are recent advances of palladium (II) complexes (4). It has been assumed that the chelating ligands will bound more effectively to the palladium(II) ion and thus imposing cis-coordination to the palladium (II) complexes of formula [ cis–pdx_2_A_2_: A_2_= a diamine or two amine. X_2_= anionic leaving ligand (s)] (5). Also, at physiological pH and ionic strength (I = 0.15), such dichloro complexes would give a variety of aqua, hydroxo, chloro or even polynuclear species depending on the compound concentration itself, as well as on the low (inside cell) and rich (blood or plasma) chloride ion concentrations. Moreover, such palladium (II) complexes can serve as good models for the understanding of more inert platinum (II) anticancer drugs (6). However, the chelated ligand may take part in processes such as acid dissociation, hydrolytic reactions and formation of insoluble complexes which are to be verified .

One of the strategies in an attempt to reduce the toxicity of cisplatin has been the use of sulphur containing ligands such as cysteine , penicillamine, methionine, thiourea, thiosulphate and particularly chelating diethyldithiocarbamate (DDTC) (7). This is because, the use of DDTC in combination with cisplatin has protected a variety of animal species from renal, gastrointestinal and bone marrow toxicities induced by cisplatin (8). More over, DDTC has remarkable property of reversing platinum binding to the macromolecules responsible for host toxicity. However, it does not interfere with the tumoricidal platinum-DNA interaction in the tumor cell. This protective action of DDTC against the toxicity of cisplatin seems to be the formation of stable Platinum-DDTC complex. Two *α*-diimine platinum (II) complexes of DDTC have also shown good antitumor activity (9). However, from the point of structural activity relationship, other derivatives of dithiocarbamates could be more interesting.

Thus, in this paper we report the synthesis, characterization and cytotoxicity of three diamine palladium (II) complexes of alkyldithiocarbamate derivatives and the effect of hydrocarbon chain length present in the structure of these complexes on cytotoxicity. Also, the cytotoxic data obtained from these complexes have been compared with cisplatin and its palladium analogue.

## Experimental

*Materials*

Butylamine, hexylamine and octylamine were bought from Merck and distilled before use. Other reagents like PdCl_2_ , NaOH, NaCl, and 2/2′- bipyridine were also purchased from Merck and used without further purification. Solvents used were reagent grade and purified before use by standard procedures. Dichloro-2,2′-bipyridinepalladium (II), [Pd (bpy) Cl_2_], cisplatin, cis-[Pt (NH­_3_)_2_ Cl_2 _] and palladium analog of cisplatin, cis- [Pd (NH­_3_)_2_ Cl_2 _] were prepared as reported earlier (10). 

Human ovarian adenocarcinoma OV2008, human hepatocarcinoma HepG2, and human lung adenocarcinoma A549 cell lines were obtained from Pharmacology lab, Ottawa Regional Cancer Center (Ottawa, Canada). DMEM/F12 medium, fetal bovine serum and trypsin and tripan blue dye were purchased from Gibco BRL, USA. 

*Methods*

Infrared spectra of ligands and metal complexes were recorded on a Nicolet 5- DXB FT-IR spectrophotometer in the range of 4000-400 cm^-1^ in KBr pellets. Electronic absorption spectra of palladium complexes were measured on a Shimadzu UV-265 recording spectrophotometer. Conductivity measurements were carried out on a Systronics conductivity bridge, model 305, with a cell (cell constant 0.59) using water conductivity as a solvent. Microchemical analysis (CHN) was done in the CHN-rapid Herause. ^1^H- NMR spectra were recorded on a Brucker DRX-500 Avance spectrometer at 500 MHz in DMSO-d_6_ using sodium 2,2- dimethyl -2- silapentane- 5- sulphonate (DSS) as internal references. Melting points were measured on a Unimelt capillary melting point apparatus and reported uncorrected. 

*Synthesis of ligands and complexes Butylamine dithiocarbamate sodium salt (Bu- dtcNa)*

A modified literature method (10) was followed for the synthesis of this compound: A mixture of butylamine (10.00 mL , 100 m mol) and NaOH (4.00 g, 100 m mol) in 80 mL acetone was stirred for 1 h. Stirring continued at 0^˚^C in an ice bath and carbon disulfide (10.00 mL excess) was added dropwise over 15 min, after which the solution become cruddy and yellow. The reaction mixture was stirred for another 5 h at 0°C and overnight at room temperature. It was then filtered, and 100 mL ether was added to the filtrate and the solution was placed in the refrigerator overnight. The resulting white precipitate of crude product was filtered off and vacuum dried. For recrystallization, the crude product was stirred with 60 mL acetone and undissolved particles were filtered out. Dichloromethan (50 mL) was added to the filterate and then left in a refrigerator overnight. The desired product was collected by filtration as white needle-like crystals and washed with small amount of dichloromethane and vacuum dried. Yield was 13.68 g (80%) with a melting point of 73°C. Analytical Calculation for C_5_H_10_NS_2_Na is as (171.12): C,35.09;H,5.85;N,8.19. Found : C, 35.02;H, 5.84; N, 8.18%.

*Hexylamine dithiocarbamate sodium salt (Hex- dtcNa)*

The synthesis procedure adopted for Bu-dtcNa was used here except that hexylamine (13.25 mL, 100 m mol) was used in place of butylamine. yield: 15.52g (78%) and melting point is 138°C. Anal. Calc. for C_7_H_14_NS_2_Na (199.36): C, 42.21;H, 7.04;N;7.04. Found: C, 42.20; H, 7.02; N, 7.05%.

*Octylamine dithiocarbamate sodium salt (Oct- dtcNa)*

The synthesis method adopted here is the same as for Bu-dtcNa, only octylamine (16.53 mL, 100 mmol) was used instead of butyamine. Yield: 17.03 g (75%) and melting point is 173°C .Anal Calc .for C_9_H_18_NS_2_Na (227.29): C, 47.58; H, 7.93; N, 6.17. Found: C, 47.57; H, 7.92; N, 6.16%.

*2,2*′*-Bipyridinebutylaminedithiocarbamatopalladium (II) Chloride. [Pd(bpy) (Bu- dtc)] Cl*

[Pd (bpy) Cl_2_] (0.333g, 1 mmol) was well suspended in 150 mL acetone by vigorous stirring for 2h and then temperature was adjusted at 40°C. To this was added dropwise Bu- dtcNa (0.205 g, 1.2 mmol) dissolved in acetone (50 mL), after which the colour of suspension was yellow. The reaction mixture was refluxed for 1 h and stirred for another 10-12 h at room temperature. It was then filtered and the volume of yellow filtrate reduced to 20 mL on a rotary evaporator. It was allowed to cool to room temperature and the yellow solid formed was filtered and washed with small amount of cold water, acetone and vacuum dried. Yield: 0.268 g (60%) and decomposes at 170°C. Anal. Calc. for C_15_ H_18_N_3_S_2_ Cl Pd (445.50): C, 40.40 ;H,4.04;N, 9.43. Found: C, 40.30; H, 4.05; N, 9.45%.

*2,2*′*-bipyridinehexylaminedithiocarbamatopalladium (II) Chloride, [Pd(bpy)(Hex- dtc)] Cl*

This compound was synthesized and purified by following the procedure as given for [Pd(bpy)(Bu-dtc)]Cl complex except that Hex-dtcNa (0.239g, 1.2 mmol) was used in place of Bu-dtcNa . Yield : 0.289 g (61%) and decomposes at 180°C. Anal. Calc. for C_17_H_22_N_3_S_2_ClPd (473.50): C, 43.08; H,4.65; N, 8.87. Found: C, 43.05; H, 4.62; N, 8.84%..

*2,2*′*-bipyridineoctylaminedithiocarbamatopalladium (II) chloride , [Pd(bpy) (Oct- dtc)] Cl*

This complex was synthesized and isolated by following the procedure as given for [Pd(bpy) (Bu-dtc)]Cl complex, except that Oct-dtcNa (0.272 g, 1.2 mmol) was used instead of Bu-dtcNa. Yield : 0.316 g (63%) and decomposes at 178°C. Anal. Calc. for C_19_H_26_N_3_S_2_ClPd (501.50): C, 45.46 ; H, 5.18; N, 8.37. Found: C, 45.42; H, 5.20; N, 8.30%.

*Cell culture experiments and clonogenic assay*

Cells were cultured in DMEM/F12 medium supplied with 10% fetal calf serum of 37°C in humified incubator with 5% CO_2_. All cellular experiments were carried out in triplicates. To examine cells’ growth in this condition, 30,000 cells per well in 6 well petridishes were seeded and growth curves were drawn for each cell line. All experiments were performed on the exponentially growing cells, which were prepared by minimum three passages of the initial seed of frozen stock.

**Table 1:** Molar conductance and electronic absorption bands of [Pd (bpy)(R-NH-CSS)]Cl coplexes in water.

**Compound**	**Molar conductance of 5×10** ^-4^ ** solution cm** ^2^ ** ohm** ^-1^ ** mol** ^-1^	**Band maxima in nm**			
		**Band I**	**Band II**	**Band III**	**Band IV**
[Pd(bpy)(Bu-dtc)]Cl	96	317(2.19)^a^	307(1.96)	248(4.71)	303(2.76)
[Pd(bpy)(Hex-dtc)]Cl	92	319(2.13)	308(2.06)	249(5.71)	201(3.57)
[Pd(bpy)(Oct)]Cl	97	318(1.88)	307.4(1.82)	247(6.50)	203(3.78)

^a^Extinction coefficients in liter mole^-1^ cm^-1^×10^-4^ are given in parentesis

For the clonogenic assay, cells from above three different cell lines were harvested with trypsin, washed with medium, and plated in quadruplicate onto 60 mm plastic tissue culture dishes at a density of 500 cells/dish in 4 mL culture medium. The cells were incubated overnight and allowed to attach on the surface of the dishes. Cells were then exposed to the various concentrations of each of above synthesized compounds, and/or cisplatin for one hour at 37°C. Three controls were used to normalize resulting data. To do so, a quadruplicate set of dishes was treated with saline, another set with DMSO solvent of these compounds as controls for experiments. The medium was then aspirated and cells were twice rinsed with saline and then the fresh medium was added to each dish. Each experiment was performed in triplicates. After 7-14 days incubation (based on the cell line growth parameters) the medium was aspirated, and cells were fixed and stained with tripan blue dye. Colonies of cells containing at least 50 cells were counted under a microscope. Percentages of colonies for each concentration compared to the appropriate control were assigned as the measurement of cytotoxicity for different concentrations.

## Results and Discussion

Three complexes of formula [Pd(bpy)(R-NH-CSS)]Cl ( Where bpy is 2,2′- bipyridine and R-NH-CSS is an anion of butylamine- , hexylamine -, and octylamine – dithiocarbamate) were prepared by interaction of [ Pd (bpy) Cl_2_] with sodium salt of dithiocarbamate in molar ratio of 1:1. The molar conductance values of these complexes in water conductivity are in the range of 91-97 cm^2 ^ohm^-1 ^mol^-1 ^(Table 1). These values suggest that they are 1:1 electrolytes (11). The chemical analysis and molar conductance data support the above formulation of the palladium complexes.

In the electronic absorption spectra of above three complexes, four bands are observed. The band maxima with their extinction coefficients are given in Table 1. The band I and II show blue shifts of 6-7 nm from less polar chloroform to more polar water. Therefore, These bands may tentatively be assigned to charge transfer from metal to 2,2′- bipyridine ligand. Band III and IV are assigned to first and higher internal transition of 2.2′- bipyridine (11).

**Figure 1:**
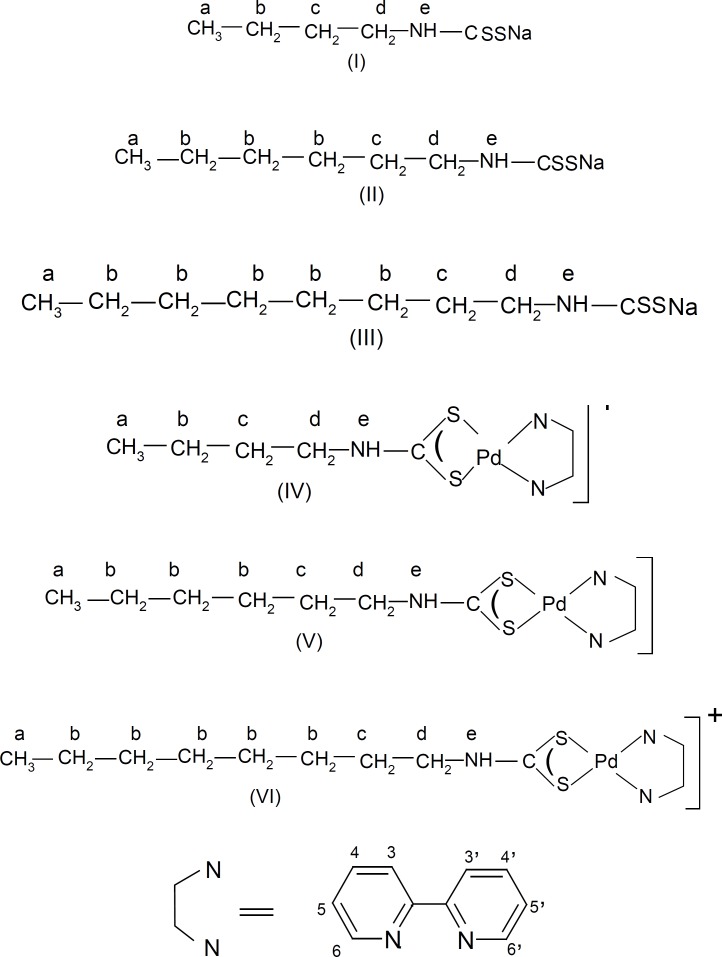
Proposed structures and numbering scheme of (1) Bu-dtcNa (2) Hex-dtcNa (3) Oct-dtcNa (4) [Pd(bpy)(Bu-dtc)]Cl (5) [Pd(bpy)(Hex-dtc)]Cl and (6) [Pd(bpy)(Oct-dtc)]Cl compounds.

Two most significant bands in the IR spectra of the ligands and the complexes are of interest; the ligands Bu-dtcNa, Hex-dtcNa and Oct–dtcNa showed a strong absorption at 1480, 1491 and 1492 cm^-1^ respectively, which is assigned to N-CSS stretching mode, while the complexes [Pd (bpy) (Bu-dtc)] Cl, [Pd (bpy)(Hex-dtc)] Cl and [Pd (bpy)(Oct-dtc)] Cl showed absorption at 1534, 1551 and 1539 cm^-1^ respectively. These absorption data suggested that the N-CSS bond order is in between a single bond (=1250-1350 cm^-1^) and a double bond (=1640-1690 cm^-1^) . Also, on passing from free dithiocarbamate ligands to corresponding complexes, the (N-CSS) mode shifted to higher energies on coordination, showing an increase in the nitrogen-carbon double bond character, caused by electron delocalization towards the palladium center. Thus the above alkyldithiocarbamate ligands coordinate to Pd (II) centers through sulfur atoms. Second, the presence of a single strong band at 923, 983 and 930 for Bu-dtcNa, Hex-dtcNa and Oct-dtcNa, respectively, and at 1026, 1027 and 1030 cm^-1^ for [Pd(bpy)(Bu-dtc)]Cl, [Pd (bpy)(Hex-dtc)]Cl and [Pd (bpy)(Oct-dtc)]Cl, respectively attributed to (SCS) mode is a strongly indicative of a completely symmetrically bonding at dithiocarbamat ligands, acting in a bidentate mode in our complexes (Figure 1). Otherwise a doublet would be expected in the 1000 ± 70 region which indicates an asymmetrically bonded ligand or a monodentate bound ligand.

The ^1^H NMR spectra of sodium salts of butylamine dithiocarbamate (Bu-dtcNa), hexylamine dithiocarbamate (Hex-dtcNa) and octyamine dithiocarbamate (Oct-dtcNa) in DMSO-d_6_ were recorded using DSS as internal reference. The ^1^H NMR spectrum of Bu-dtcNa shows five peaks at 0.82, 1.21, 1.39, 3.31 and 8.08 ppm which are assigned to three H-a, Two H-b, two H-c two H-d and one H-e protons respectively (Table 2 and figure 1-I). Similar assignments have been done for analogous ligands Hex-dtcNa and Oct-dtcNa which data are collected in Table 2. The integrated areas under the peaks correspond to the ratio 3 : 2 : 2 : 2 : 1 for Bu-dtcNa, 3 : 6 : 2 : 2 : 1 for Hex-dtcNa and 3 : 10 : 2 : 2 : 1 for Oct-dtcNa and thus support the proposed structures.

**Table 2:** ^1^H NMR spectral data of R-NH-CSSNa ligands and [Pd(bpy)(R-NH-CSS)]Cl complexes in deuterated DMSO.

**compounds**	**2,2΄-bipyridine protons**				**Dithiocarbamate proton**				
	**H-6,6΄**	**H-4,4΄**	**H-3,3΄**	**H-5,5΄**	** H-a**	**H-b**	**H-c**	**H-d**	**H-e**
**Bu-dtcNa**					0.82 (t)	1.21(m)	1.39 (m)	3.31 (m)	8.08 (sb)
**[Pd(bpy)(But-dtc)]Cl**	8.50^α^(d)^β^	8.25(t)	8.06(q)	7.64(t)	0.86 (m)	1.29 (m)	1.55 (m)	3.37 (m)	11.51 (sb)
**Hex-dtcNa**					0.84 (m)	1.23 (m)	1.41(m)	3.31 (m)	7.98 (sb)
**[Pd(bpy)(Hex -dtc)]Cl**	8.54(d)	8.32(t)	8.22(q)	7.71(t)	0.83 (m)	1.23 (m)	1.54 (m)	3.40 (m)	11.59 (sb)
**Oct-dtcNa**					0.84 (t)	1.23 (m)	1.41(m)	3.31 (m)	7.98 (sb)
**[Pd(bpy)(Oct-dtc)]Cl**	8.51(d)	8.26(t)	8.15(q)	7.69(m)	0.82 (m)	1.24 (m)	1.57 (m)	3.45 (m)	11.53 (sb)

^α ^chemical shift in ppm.^ β ^s,d,t,q,m and sb are singlet , doublet , triplet, quartet, multiplet and singlet broad , respectively.

The ^1^H NMR spectra of [Pd (bpy(R-NH-CSS)]Cl complexes were recorded in deuterated dimethyl sulphoxide (DMSO-d_6_) using DSS as internal reference. Their chemical shifts along with the coupling constants are summarized in Table 2 and proposed structure and NMR numbering scheme are given in Figure 1. The protons of 2,2′-bipyridine moieties in [Pd (bpy)(Bu-dtc)] Cl appear as a doublet at 8.50 ppm, a triplet at 8.25 ppm, quartet at 8.06 ppm and triplet at 7.64 ppm which are assigned to H-6,6′, H-4,4′, H-3,3′ and H-5,5′ protons, respectively (10). similar assignements for protons of 2,2′-bipyridine moieties in [Pd (bpy)(Hex-dtc)] Cl and [Pd(bpy)(Oct-dtc)]Cl are given in Table 2. In the [Pd(bpy) (Bu-dtc)]Cl Complex two multiplets observed at 0.86 and 1.99ppm are assigned to three H-a and two H-b protons (Figure 1). The two other multiplets appeared at 1.55 and 3.37 ppm is assigned to H-c and H-d protons of dithiocarbamate moieties. Another broad singlet (possibly multiplet) resonates at 11.51 ppm is assigned to H-e proton of coordinated dithiocarbamate. This peak disappear on D_2_O exchange and also shows an upfield shift of 3.35 ppm in the complex as compared to its value in sodium dithiocarbamate. This suggests the bonding of dithiocarbamate ligand to palladium (II) through both sulphur atoms. Similar assignements have been done for protons of dithiocarbamate moieties in the analogous complexes [Pd(bpy)(Hex-dtc)]Cl and [Pd(bpy)(Oct-dtc)]Cl (Table 2). The integrated areas under the peaks of 2,2′-bipyridine and dithiocarbamate protons in the ratio 8 : 10 for[Pd (bpy)(Bu-dtc)]Cl and 8:14 for [Pd(bpy)(Hex-dtc)]Cl and 8 : 18 for [Pd (bpy)(Oct-dtc)]Cl are further supporting the proposed structures. 

Finally,^ 1^H NMR spectra of the complexes dissolved in DMSO-d_6_ and recorded after 24 h observed no changes in these spectra, suggesting no dissociation of dithiocarbamate anions.

**Table 3:** Cytotoxicty in LD_50_ of synthesized Pd compounds (abbreviations as per Figure 1), as well as cisplatin and its Pd analogue on three different human cancer cell lines in µg/mL.

**CELL LINE** **COMPOUND**	**A549**	**HepG2**	**OV2008**
IV	0.379 ± 0.107	0.934 ± 0.194	0.234 ± 0.023
V	0.605 ± 0.054	0.131 ± 0.052	0.914 ± 0.097
VI	0.482 ± 0.152	0.172 ± 0.060	0.258 ± 0.043
Pd Analogue	1.721 ± 0.793	8.833 ± 0.729	1.53 ± 0.557
cisplatin	2.799 ± 0.733	2.196 ± 0.220	0.838 ± 0.074

DNA binding and antitumor activities of these compounds on human K562 cell line have been published before (10). Here, using the same derivatives, we have examined the cytotoxicity effects of above mentioned compounds on three different human cell lines as potential candidates for human cancer therapy with the results shown in Table 3. In this table, clonogenic results (mean ± SEM) are listed as the percentages of colonies survived after exposure to different concentrations of compounds in comparison with the control. As is shown in this table from the clonogenic assay, a typical dose-response curve with LD_50_s of 0.838 ± 0.074 µg/mL, 2.196 ± 0.220 µg/mL and 2.799 ± 0.733 µg/mL, have been shown on OV2008, HepG2 and A549, respectively for cisplatin. The rank order of cisplatin cytotoxicity on these cell lines is in well agreement with the previous publication and the cell lines sensitivities to this anticancer drug. This may be counted as a proof for the accuracy of cytotoxicity assay. As is shown in Table 3, the Pd analogue of cisplatin is not following the same ranking order of cytotoxicity on these cell lines, being most toxic for OV2008 and A549 and less for the HepG2. This compound may be a good candidate for further studies as a potential anticancer drug for the lung adenocarcinoma which is considered as a very resistant cancer to chemotherapy. Other synthetic Pd compounds are in the same cytotoxic range as cisplatin for different cell lines, but with a distinguished effect on lung adenocarcinoma. Above biological study may suggest these Pd analogues of cisplatin as good candidates to be considered for the treatment of human lung cancer. Further cellular and animal studies are suggested aiming toward the potential clinical application of these compounds.
